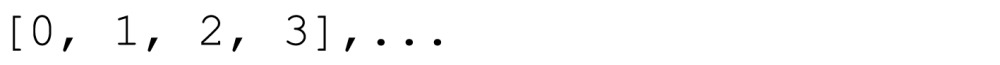# Integration of TomoPy and the ASTRA toolbox for advanced processing and reconstruction of tomographic synchrotron data

**DOI:** 10.1107/S1600577516005658

**Published:** 2016-04-28

**Authors:** Daniël M. Pelt, Doǧa Gürsoy, Willem Jan Palenstijn, Jan Sijbers, Francesco De Carlo, Kees Joost Batenburg

**Affiliations:** a Centrum Wiskunde and Informatica, Science Park 123, 1098 XG Amsterdam, The Netherlands; b Advanced Photon Source, Argonne National Laboratory, 9700 South Cass Avenue, Argonne, IL 60439-4837, USA; c iMinds–Vision Lab, University of Antwerp, Universiteitsplein 1, B-2610 Antwerp, Belgium; d Mathematical Institute, Leiden University, Niels Bohrweg 1, 2333 CA Leiden, The Netherlands

**Keywords:** tomography, TomoPy, ASTRA toolbox

## Abstract

The processing of tomographic synchrotron data requires advanced and efficient software to be able to produce accurate results in reasonable time. In this paper, the integration of two software toolboxes, TomoPy and the ASTRA toolbox, which, together, provide a powerful framework for processing tomographic data, is presented. The integration combines the advantages of both toolboxes, such as the user-friendliness and CPU-efficient methods of TomoPy and the flexibility and optimized GPU-based reconstruction methods of the ASTRA toolbox. It is shown that both toolboxes can be easily installed and used together, requiring only minor changes to existing TomoPy scripts. Furthermore, it is shown that the efficient GPU-based reconstruction methods of the ASTRA toolbox can significantly decrease the time needed to reconstruct large datasets, and that advanced reconstruction methods can improve reconstruction quality compared with TomoPy’s standard reconstruction method.

## Abstract 2

The integration of two Python toolboxes used for processing tomographic data, TomoPy and the ASTRA toolbox, is presented.

## 1. Introduction   

In transmission X-ray tomography experiments performed at synchrotron facilities, large amounts of projection data are produced in a short time. Current detector technology allows one to collect projections at kHz frame rate, enabling three-dimensional imaging of dynamic systems (Gibbs *et al.*, 2015 ▸), *in situ* studies of materials (Patterson *et al.*, 2016 ▸) and monitoring the evolution of biological systems (Moosmann *et al.*, 2013 ▸).

Processing these datasets in a time comparable with data collection is essential to properly capture the sample evolution and adjust the instrument settings during the experiment; this requires algorithms optimized for high-performance computing (HPC), which have to be easily available and usable by the beamline users. Furthermore, many advanced experiments, such as those with extremely high spatial or temporal resolutions (Sakdinawat & Attwood, 2010 ▸; Mokso *et al.*, 2013 ▸) and of dose-sensitive objects (Lovric *et al.*, 2013 ▸), require a variety of pre-processing, post-processing and reconstruction algorithms to reduce artifacts in the final reconstruction.

In this paper, we present the integration of two Python toolboxes which, together, allow users to easily apply advanced tomographic algorithms on large-scale experimental datasets in an efficient way: the TomoPy toolbox (Gürsoy *et al.*, 2014 ▸) and the ASTRA toolbox (van Aarle *et al.*, 2015 ▸). By combining both toolboxes, we are able to leverage the advantages of both to create an improved workflow for beamline users.

The TomoPy toolbox is specifically designed to be easy to use and deploy at a synchrotron facility beamline. It supports reading many common synchrotron data formats from disk (De Carlo *et al.*, 2014 ▸), and includes several pre-processing and post-processing algorithms commonly used for synchrotron data. TomoPy also includes several reconstruction algorithms, which can be run on multi-core workstations and large-scale computing facilities. The algorithms in TomoPy are all CPU-based, however, which can make them prohibitively slow in the case of iterative methods, which are often required for advanced tomographic experiments.

The ASTRA toolbox provides highly efficient tomographic reconstruction methods by implementing them on graphic processing units (GPUs). It includes advanced iterative methods and allows for very flexible scanning geometries. The ASTRA toolbox also includes building blocks which can be used to develop new reconstruction methods, allowing for easy and efficient implementation and modification of advanced reconstruction methods. However, the toolbox is only focused on reconstruction, and does not include pre-processing or post-processing methods that are typically required for correctly processing synchrotron data. Furthermore, no routines to read data from disk are provided by the toolbox.

By integrating the ASTRA toolbox in the TomoPy framework, the optimized GPU-based reconstruction methods become easily available for synchrotron beamline users, and users of the ASTRA toolbox can more easily read data and use TomoPy’s pre-processing and post-processing methods.

This paper is structured as follows: in §2, we give a more detailed explanation of TomoPy and the ASTRA toolbox, and explain how we integrated them. In §3, we give general instructions on how to install and use the combined toolboxes in practice. An example for a specific dataset in given in §4, and we conclude the paper in §5.

## 2. Integrating TomoPy and the ASTRA toolbox   

### 2.1. TomoPy   

TomoPy is an open-source Python toolbox to perform tomographic data processing and image reconstruction tasks, developed at the Advanced Photon Source of Argonne National Laboratory (Gürsoy *et al.*, 2014 ▸). The toolbox is available for Linux and OS X operating systems, and is aimed at providing a high-level interface for processing and tomographic reconstruction of datasets at synchrotron light sources. TomoPy relies on standard scientific packages like NumPy, SciPy and Scikit, and offers a free, open-source, modular, readable and manageable framework that researchers can use and contribute to easily. Python also offers easy integration with C or Fortran code through shared libraries in situations where computation speed is critical. In addition, the native control software running at several synchrotron facilities, EPICS (http://www.aps.anl.gov/epics), is accessible *via* Python (http://pyepics.github.io/pyepics/), allowing simultaneous data analysis and real-time feedback on the instrumentation status. So far, TomoPy has been employed in reconstructions for various techniques from micro-CT (Duke *et al.*, 2015 ▸) to X-ray fluorescence tomography (Gürsoy *et al.*, 2015*a* ▸), X-ray scattering tomography (Gürsoy *et al.*, 2015*b* ▸), Lorentz electron microscopy (Phatak & Gürsoy, 2015 ▸) and deployed on large-scale computing facilities (Biçer *et al.*, 2015 ▸).

TomoPy includes a plethora of processing functions from pre-processing to image reconstruction of synchrotron tomographic data. It includes ring removal algorithms, such as the generalized Titarenko’s algorithm (Miqueles *et al.*, 2014 ▸) and a Fourier wavelet approach (Münch *et al.*, 2009 ▸), and a zinger correction algorithm based on median filters. The estimation of the rotation center can be calculated using the image entropy calculation based method (Donath *et al.*, 2006 ▸) or Vo’s Fourier method (Vo *et al.*, 2014 ▸). A single-step X-ray phase-retrieval algorithm based on Paganin filtering is available for phase-contrast datasets (Paganin *et al.*, 2002 ▸). TomoPy also includes algorithms for post-processing reconstructed images, such as Gaussian filtering and median filtering to reduce noise artifacts.

In addition to Gridrec (Dowd *et al.*, 1999 ▸), which is the traditionally used analytical image reconstruction algorithm, TomoPy also offers variants of algebraic reconstruction methods (ART, BART, SIRT) (Kak & Slaney, 2001 ▸), and maximum-likelihood expectation maximization (ML-EM) approaches (Dempster *et al.*, 1977 ▸), as well as their regularized variations (PML) (Chang *et al.*, 2004 ▸). Ordered-subset implementation of all algorithms are also available for efficient calculations; for example, the well known ordered-subset expectation maximization (OSEM) algorithm (Hudson & Larkin, 1994 ▸). An overview of all algorithms included in TomoPy, along with their parameters and usage examples, can be found on the documentation website of TomoPy (http://tomopy.readthedocs.org).

Another important property of TomoPy is that it provides X-ray matter interaction simulation tools, such as X-ray transmission or wave propagation, that can be used to evaluate the efficiency of various coding scenarios or as a platform for modeling. The standard installation package of TomoPy is optimized for use at a workstation, but TomoPy algorithms are also suitable for grid-computing and massive parallelization when needed. Experiments with MPI implementations of iterative algorithms and tomography datasets with 1K projections of 2K by 2K pixels show that TomoPy’s iterative methods can scale up to thousands of cores on an IBM BG/Q supercomputer with almost perfect speedup and can reduce total reconstruction times for such datasets by more than 95.4% on 32K cores relative to 1K cores. Moreover, the average reconstruction times are improved from 2 hours (256 cores) to 1 minute (32K cores), thus enabling near-real-time use (Biçer *et al.*, 2015 ▸).

### 2.2. The ASTRA toolbox   

The ASTRA toolbox is an open-source software toolbox developed at the University of Antwerp, Belgium, and at the Centrum Wiskunde & Informatica (CWI), Amsterdam, The Netherlands, that is focused on the reconstruction of two-dimensional (slice-based) and three-dimensional tomographic datasets (van Aarle *et al.*, 2015 ▸). The toolbox is available for Linux and Windows operating systems, and is aimed at providing a fast and flexible development platform for tomographic reconstruction algorithms. Because of its flexibility, it can be applied to various scanning geometries and acquisition modes, such as (bio)medical and industrial µCT (Plantagie *et al.*, 2015 ▸), electron tomography (Roelandts *et al.*, 2012 ▸), neutron tomography (Peetermans & Lehmann, 2013 ▸; Van Eyndhoven *et al*., 2015 ▸) and synchrotron tomography (Reischig *et al.*, 2013 ▸). The toolbox uses CUDA for NVIDIA GPUs to perform accelerated parallel computations, reducing the computation time of many tomographic operations (Palenstijn *et al.*, 2011 ▸). Most two-dimensional slice-based operations can also be run on standard CPUs, in which case the toolbox supports different projection kernels, *i.e.* ways of discretizing the projection operations. A comparison of various projection kernels can be found in the paper by Xu & Mueller (2006 ▸). When using GPUs, only a single set of projection kernels is supported for optimal performance. Through either a MATLAB or Python interface, the tomographic operations can be easily used and combined with other numerical code or software for pre-processing, post-processing or analysis of the acquired data. The toolbox also provides a matrix-like interface to linear tomography operators, allowing them to be easily used in existing and new code (Bleichrodt *et al.*, 2015 ▸). A version of the ASTRA toolbox with MPI support for high-performance computing is available as a separate package (Palenstijn *et al.*, 2015 ▸).

The ASTRA toolbox includes many popular tomographic reconstruction methods (see Table 1 ▸), such as the analytic filtered backprojection (FBP) method and the iterative SIRT method (Kak & Slaney, 2001 ▸) and CGLS method (Hansen, 1998 ▸). These methods support various parameters that can help improve reconstruction quality; for example, the choice of filter to use in the FBP method, and additional nonnegativity constraints in the SIRT method. An important feature of the ASTRA toolbox is that it also provides *building blocks* that can be used to develop advanced tomographic reconstruction methods. For example, using the optimized methods for the forward projection of objects and the backprojection of sinograms, it is possible to develop efficient advanced iterative methods, such as total variation regularized methods, using the ASTRA toolbox. A recent addition to the toolbox is a *plugin* system, which enables algorithm developers to easily distribute new tomographic reconstruction methods, and ASTRA users to easily install and use them with minimal changes in production scripts. With the TomoPy integration presented in this paper, these ASTRA plugins will automatically be usable in TomoPy as well.

### 2.3. Implementation   

The code to integrate TomoPy and the ASTRA toolbox is written in the Python language, since TomoPy is mainly written in Python and the ASTRA toolbox includes a Python interface as well. Specifically, a first step was to add code to TomoPy that enables the use of other Python libraries to perform tomographic reconstruction instead of TomoPy’s included algorithms. Using this new feature, code was added which enables the use of the ASTRA toolbox to perform the reconstruction. By integrating the ASTRA toolbox into TomoPy in this way, synchrotron users that are familiar with TomoPy will be able to use the ASTRA toolbox with minimal effort. Note that other tomographic reconstruction libraries that include a Python interface can be integrated in TomoPy in the same way. Since the integration requires both toolboxes to be installed on the same machine, it is only supported on operating systems in which *both* TomoPy and the ASTRA toolbox can be installed. Currently, this is only the case for modern Linux operating systems, but other operating systems will be supported once both TomoPy and the ASTRA toolbox support them.

**Figure 1:**
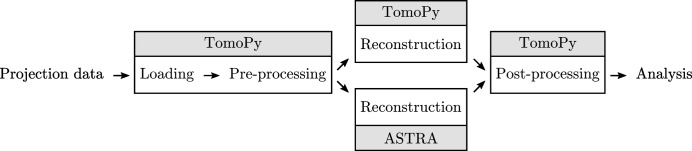
Schematic overview of the workflow of processing a dataset with the integrated TomoPy and ASTRA framework. Note that the reconstruction step can be performed by either TomoPy or the ASTRA toolbox.

In the interfacing code, the parallel-beam geometry defined by TomoPy (*i.e.* the number of detector pixels, the angles for which projections are acquired, and the center of rotation) is translated to a corresponding ASTRA geometry, and the chosen ASTRA reconstruction method is performed. Afterwards, the result of the reconstruction is stored in TomoPy memory, and all ASTRA objects are cleaned up. In this way, the reconstruction step is completely self-contained and independent of any pre-processing or post-processing step. An advantage of this independence is that user scripts do not have to be rewritten to use the ASTRA toolbox: only the reconstruction function call has to be modified. Also, changing between different reconstruction methods, between CPU and GPU implementations, and between different reconstruction parameters usually only requires changes in a single line of the user script. Examples of these minimal changes are shown in §3.2. A schematic overview of the full processing workflow from loading the raw data to analysis is shown in Fig. 1 ▸.

## 3. Installation and usage   

### 3.1. Installation   

Both TomoPy and the ASTRA toolbox can be installed using the Conda package management system (http://conda.pydata.org). The advantage of using Conda over other Python package management systems is that Conda allows for the inclusion of non-Python library dependencies, which are commonly needed for numerical toolboxes such as TomoPy and the ASTRA toolbox. Since the goal of both toolboxes is to be easily installable at the various workstations and computational clusters available at synchrotrons, which may each be running a different environment of installed libraries and library versions, the ability to tightly control the library dependencies is important to create a user-friendly installation process. To install TomoPy in a Conda environment, the following command can be used:
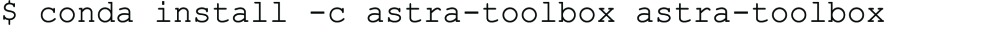


A similar command can be used to install the ASTRA toolbox:
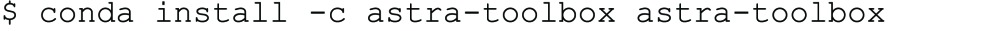


Note that both toolboxes can be installed and used independently from each other. TomoPy will automatically detect whether the ASTRA toolbox is available, and enables the use of ASTRA methods if this is the case. Both toolboxes can also be compiled and installed from source code, which can be downloaded from their respective git repositories (https://github.com/tomopy/tomopy and https://github.com/astra-toolbox/astra-toolbox). Compared with installing using Conda, it is easier to make modifications and contribute to the development of the toolboxes when installing from source code, but the compilation step requires the availability of several library dependencies on the workstation.

### 3.2. Usage   

We will now show how the new features can be used after installation of both toolboxes. The following example script shows a simple standard TomoPy workflow, loading data from disk, normalizing the data using the flatfield and darkfield images, and finally reconstructing with the standard TomoPy gridrec method (all scripts shown in this paper are available in the supporting information):
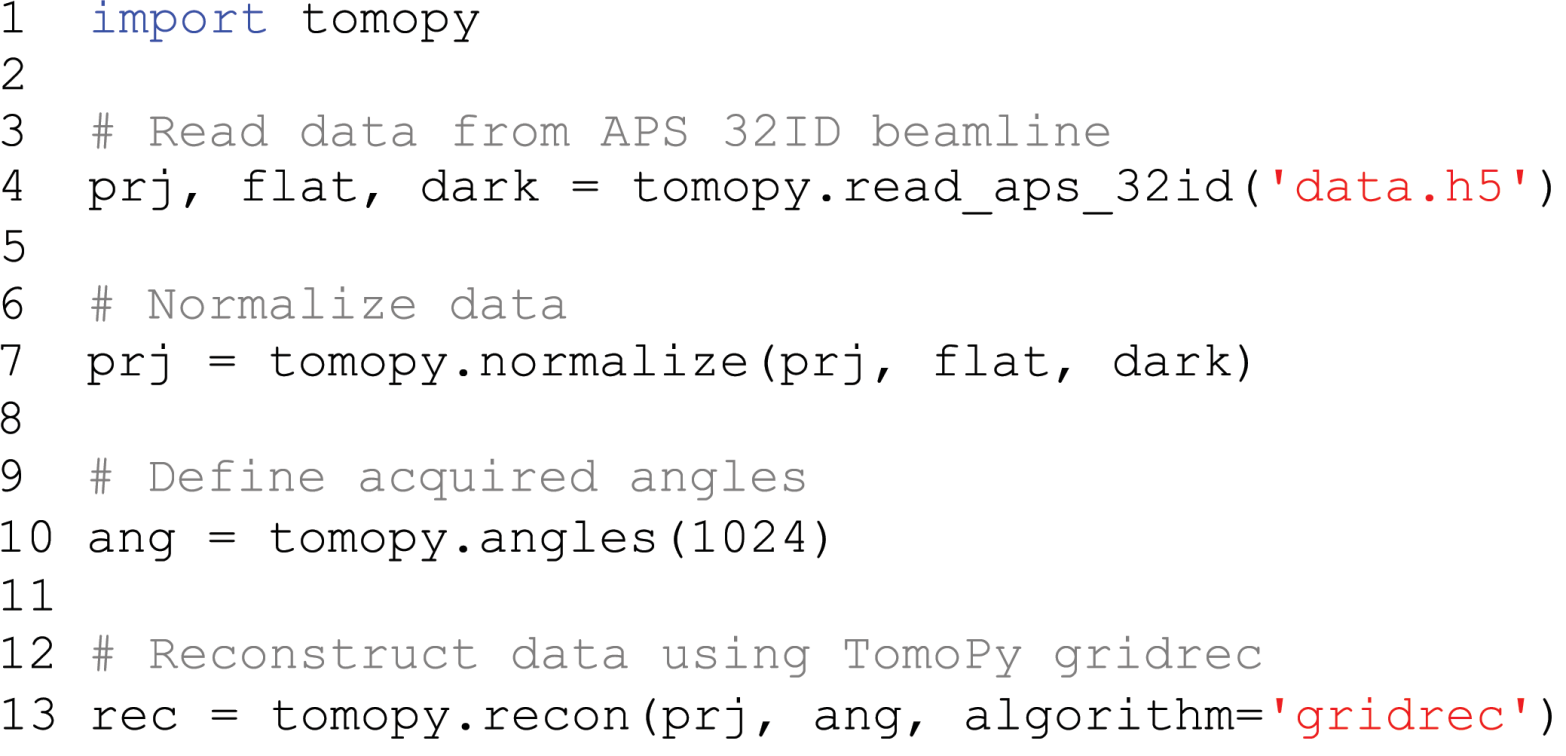


To modify this script to reconstruct using the ASTRA toolbox instead, only line 13 has to be changed, replacing 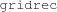 with 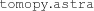, specifying which method to reconstruct with in the 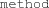 option, and specifying which type of projection kernel to use in the 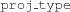 option. An overview of common options that are used when reconstructing with the ASTRA toolbox is given in Table 2 ▸. For example, to reconstruct with FBP using a voxel-driven kernel, we change the final part of the above script to:
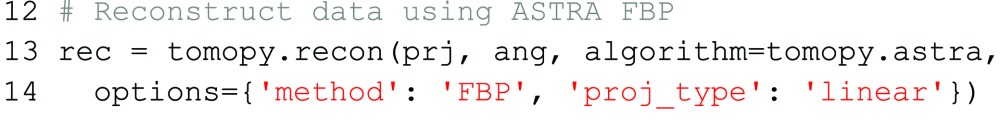


When running on a machine with a GPU with CUDA capabilities, the same reconstruction can be performed using optimized GPU code, greatly decreasing the needed computation time. This can be realised by specifying 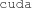 as the projection kernel, and use a GPU-enabled method (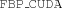):
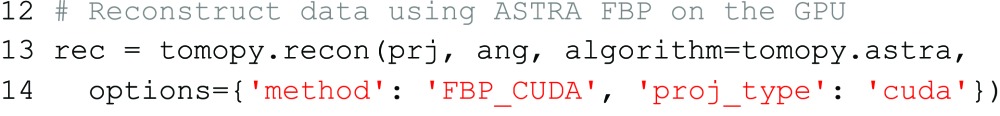


Iterative methods can be used by specifying the corresponding ASTRA method (*e.g.* 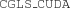 for a GPU-enabled CGLS method), and the number of iterations to use in the 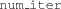 option:
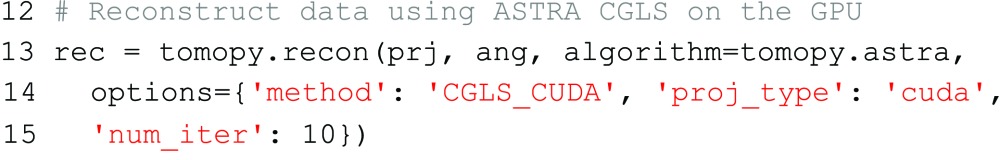


Most reconstruction methods in the ASTRA toolbox support several parameters that can help improve reconstruction quality. In the TomoPy integration, these parameters are specified by supplying them in the 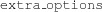 setting. For example, to add a nonnegativity constraint to the GPU-enabled SIRT method, we add 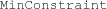 to the 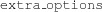 setting (note that lower bounds other than zero can also be used):
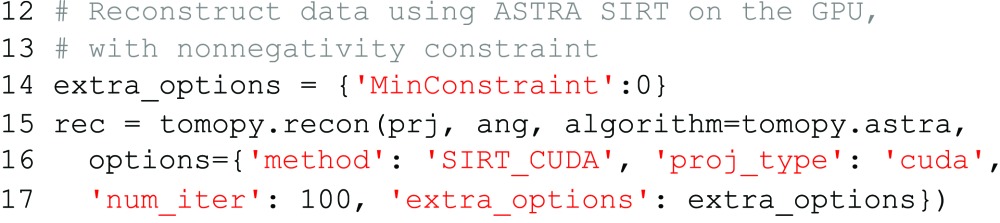


An overview of the various parameters that are supported by the reconstruction methods can be found on the website of the ASTRA toolbox (http://www.astra-toolbox.com).

If multiple GPUs are installed in the workstation running TomoPy and the ASTRA toolbox, the computations can be distributed over multiple GPUs by specifying a list of GPU indices in the 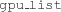 option. Since each slice of the reconstruction can be computed independently from the other slices, a significant reduction of computation time can be achieved by distributing the computations in this way. For example, to use four installed GPUs, labeled 0 through 3, we use 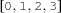 as the 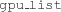:
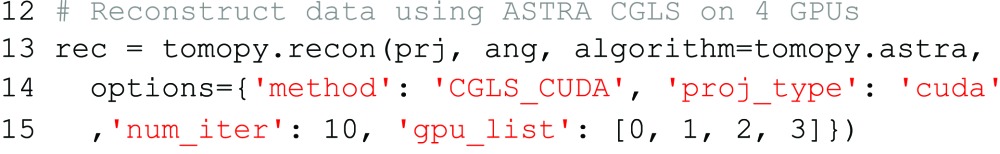


Finally, ASTRA plugins can be used by first registering them with the ASTRA toolbox itself, and using the method name defined by the plugin as the 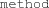 option. Extra parameters for the reconstruction can be specified using the 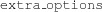 setting, similar to standard ASTRA methods. An ASTRA plugin is typically distributed as a Python class within a Python package, which has to be imported separately. After importing, the 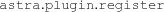 method is used to register a plugin with the ASTRA toolbox. For example, suppose that there is a plugin class 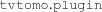 within the 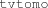 package, with the method name 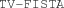 and an additional parameter 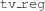. To use this plugin in TomoPy, the following code can be used:
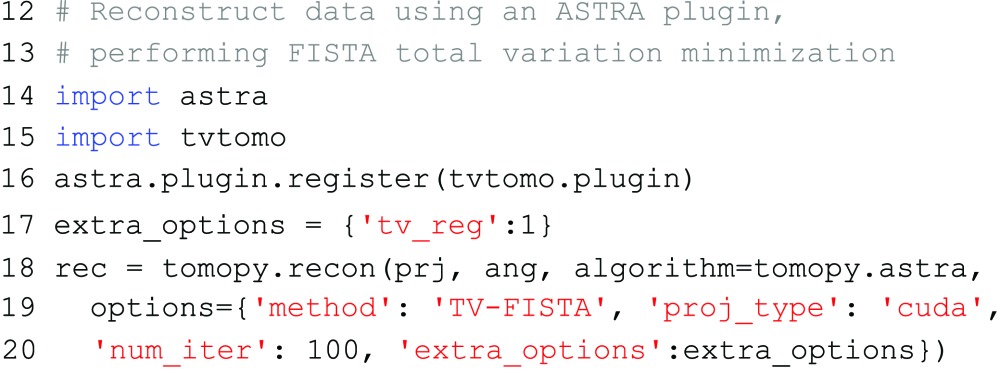


### 3.3. Computation time   

**Figure 2:**
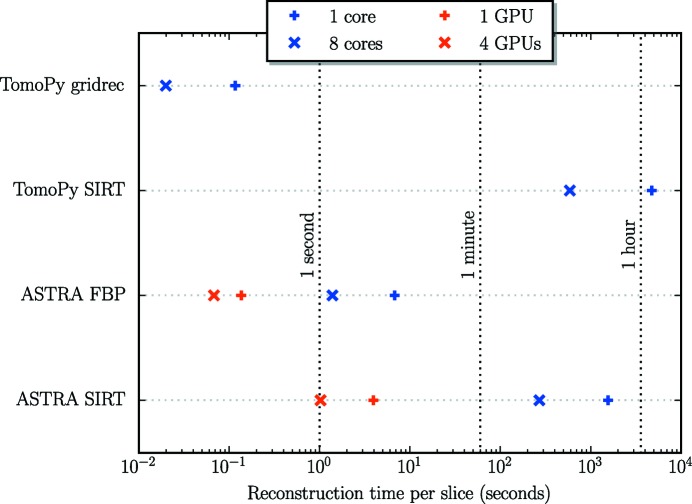
Computation time per slice of reconstructing with the gridrec method computed with TomoPy, the FBP method computed with the ASTRA toolbox, and 100 iterations of the SIRT method computed with both, for 1024 projections and a detector width of 1200 pixels. Results are shown for using a single CPU core, 8 CPU cores, a single GPU, and 4 GPUs.

In Fig. 2 ▸, a comparison is shown between the computation times per slice of reconstructions computed with different methods of both TomoPy and the ASTRA toolbox, for a single slice of a dataset with 1200 detector pixels and 1024 projections. All reconstructions were computed on a workstation with 128 GB of memory, two Intel Xeon E5-2623 v3 CPUs (four cores each, running at 3.0 GHz) and two Geforce GTX Titan Z cards (running at their default clock speeds). Each Titan Z card contains two GPUs, with 15 multiprocessors (2880 cores) and 6 GB of memory per GPU. The workstation was using the Fedora 21 operating system, with the official CUDA 7.0 drivers provided by NVIDIA. We compare the computation times of gridrec computed using TomoPy, FBP computed using the ASTRA toolbox, and 100 iterations of SIRT computed using both TomoPy and the ASTRA toolbox. For the CPU-based methods of TomoPy and the ASTRA toolbox, computation times are shown for using both a single core and all eight cores of the machine. For the GPU-based methods of the ASTRA toolbox, computation times are shown for using both a single GPU and all four installed GPUs.

The results of Fig. 2 ▸ show that for the SIRT method a significant reduction of computation time can be achieved by using GPUs instead of CPUs for computation, with the computation time per slice when using TomoPy and eight CPU cores being roughly 600 times the computation time when using the ASTRA toolbox and four GPUs. Note that this reduction of computation time can be important in practice, since, in this case, computing the SIRT reconstruction of 200 slices would take less than 4 minutes with four GPUs, compared with more than 32 hours with eight CPU cores. Therefore, by using GPU-based methods, it is possible to compute iterative reconstructions during experiments, enabling direct inspection of the reconstructions and the possibility of making adjustments to improve the experimental results during the experiment itself.

In contrast to the iterative SIRT method, TomoPy’s CPU-based gridrec method takes less time to compute compared with the GPU-based FBP method, especially when using multiple CPU cores. This is expected for problems with a relatively large number of projections, since the most costly computations of the gridrec method are the two-dimensional Fourier transforms, for which the computation time is independent of the number of projections. On the other hand, in the FBP method the most costly computation is the backprojection operation, for which the computation time scales linearly with the number of projections. Note that the reconstruction quality of gridrec and FBP reconstructions are usually similar (Marone & Stampanoni, 2012 ▸).

## 4. Example   

In this section, we give an example of the full processing workflow of reconstructing a tomographic synchrotron dataset, acquired at the 32-ID beamline of the Advanced Photon Source of Argonne National Laboratory. We compare the resulting reconstructions of a single slice using various reconstruction methods, both with and without ring-removal pre-processing applied. The dataset is of a sample under pressure in a diamond anvil cell, whose frame blocks part of the acquired projections, rendering them unusable. The result is a limited-angle tomographic problem, where the acquired projections do not span the entire 180° range. Specifically, projections of 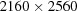 pixels were acquired in 0.5° intervals over a 137° range, for a total of 273 projections. It is typically difficult to obtain accurate reconstructions for limited-angle problems, with standard methods producing wedge artifacts in the direction of the missing projection angles (Delaney & Bresler, 1998 ▸).

**Figure 3:**
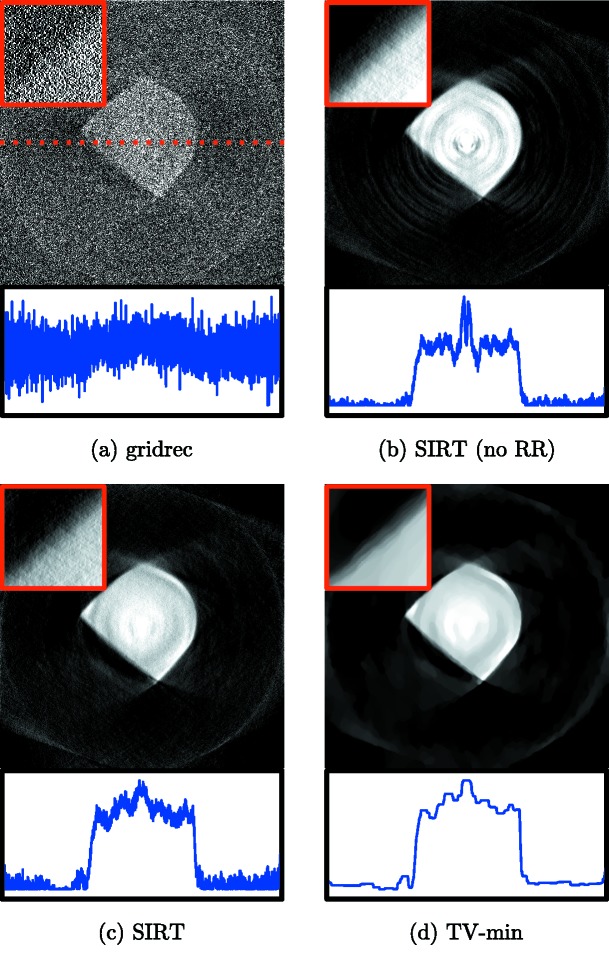
Reconstructions of a single slice of a sample in a high-pressure diamond anvil cell which blocks 86 of the 359 projections over 180°, with 2560 detector pixels per projection. Reconstructions are computed with (*a*) gridrec (TomoPy), (*b*) and (*c*) SIRT with a nonnegativity constraint (ASTRA), and (*d*) TV-minimization using FISTA (ASTRA plugin). In (*a*), (*c*) and (*d*), a ring-removal pre-processing step (Münch *et al.*, 2009 ▸) was applied using TomoPy. A line profile of the center, indicated in (*a*) by a dotted line, is shown for each reconstruction, as well as a cropped 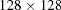 pixel section of the upper left part of the sample.

In Fig. 3 ▸, reconstructions are shown of a single slice of the sample, reconstructed with various reconstruction methods. In each reconstruction except for the one shown in Fig. 3(*b*) ▸, a ring-removal pre-processing method that is included in TomoPy (Münch *et al.*, 2009 ▸) was used to suppress ring artifacts. In Fig. 3(*a*) ▸, the reconstruction computed with TomoPy’s gridrec method is shown. In Figs. 3(*b*) and 3(*c*) ▸, ASTRA’s GPU-enabled SIRT method was used to compute the reconstructions. Finally, a reconstruction regularized with total variation minimization is shown in Fig. 3(*d*) ▸, computed using an ASTRA plugin that implements the FISTA method (Beck & Teboulle, 2009 ▸). The Python script used to compute the reconstruction of Fig. 3(*c*) ▸ is given below. Note that the scripts used to compute the other reconstructions are similar, only requiring minimal changes like the ones given in §[Sec sec3.2]3.2:
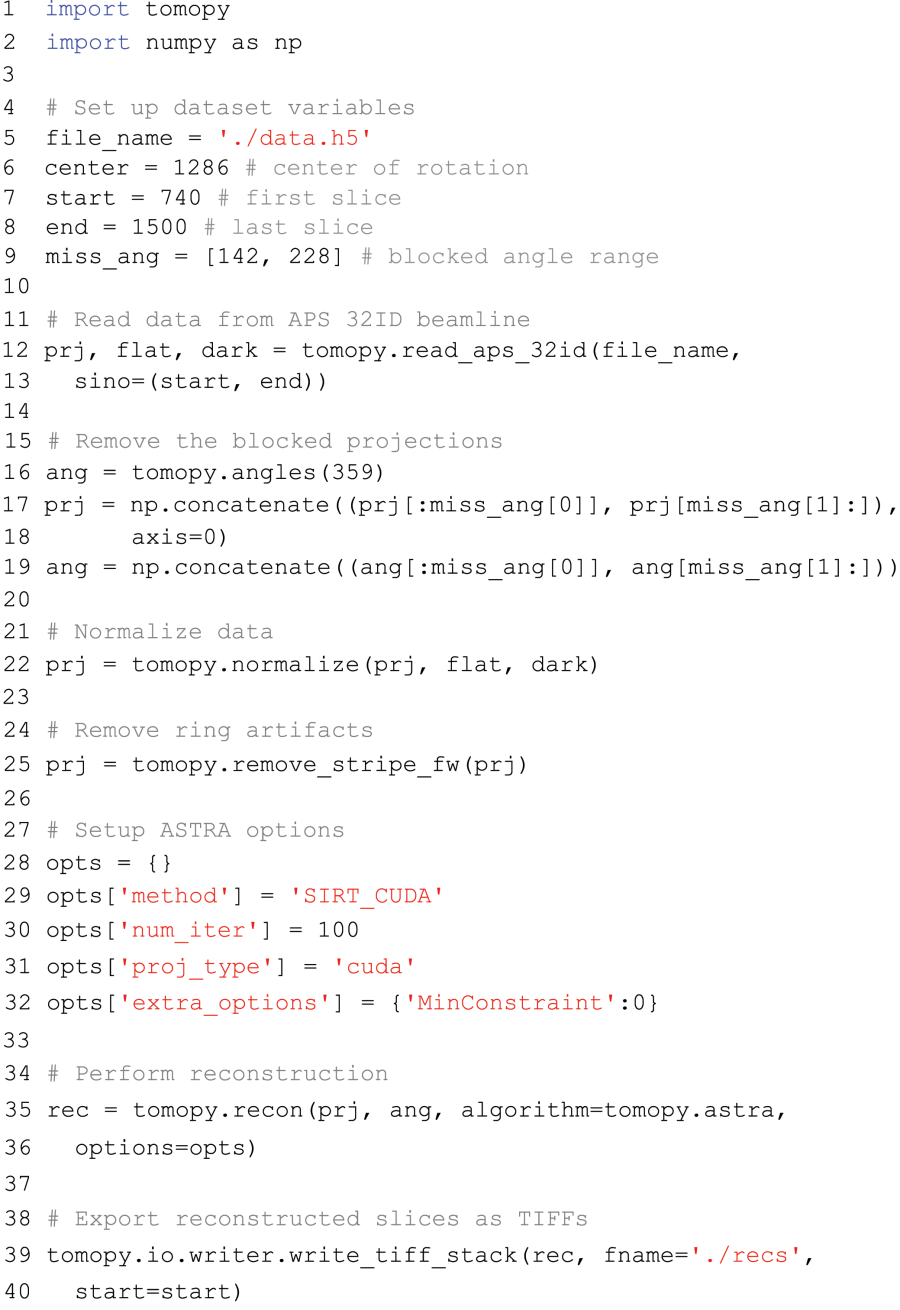


The results of Fig. 3 ▸ show that the gridrec reconstruction includes large amounts of noise artifacts, especially visible in the line profile, as well as wedge artifacts resulting from the missing projection angles. These artifacts can make further analysis, such as volume estimation, difficult or impossible, even with further post-processing. The iterative reconstructions, which can be computed efficiently using the GPU-enabled methods of the ASTRA toolbox, include fewer artifacts, significantly reducing noise in the reconstructed image. Note, however, that significant ring artifacts are present in Fig. 3(*b*) ▸, where we did not use one of TomoPy’s ring-removal pre-processing methods. In the reconstruction of Fig. 3(*c*) ▸, the ring artifacts are significantly reduced, which shows that TomoPy’s advanced pre-processing methods can be used to improve the reconstruction quality of ASTRA’s reconstruction methods. Finally, the total variation minimization reconstruction of Fig. 3(*d*) ▸ shows that advanced regularized reconstruction methods can be distributed as ASTRA plugins and be used in combination with TomoPy to minimize artifacts in the final reconstruction.

## 5. Conclusions   

In this paper, we presented the integration of two Python toolboxes used for processing tomographic data: TomoPy and the ASTRA toolbox. The integration allows for combining the advanced I/O, pre-processing and post-processing capabilities of TomoPy with the advanced tomographic reconstruction methods of the ASTRA toolbox. One advantage of the integration is that it enables the use of GPU-enabled methods included in the ASTRA toolbox to significantly improve computation time, especially for iterative reconstruction methods. Another advantage is that advanced iterative methods can be written and distributed as ASTRA plugins and subsequently used within TomoPy. Code has been added to TomoPy that automatically creates the necessary ASTRA objects, cleaning them up after computation has finished. As a result, only minimal changes are needed in user scripts to use the ASTRA toolbox within TomoPy.

We have shown how to install both toolboxes on a single machine, and how to use the various features of the integrated software. In particular, we have shown how to adjust an existing TomoPy script to reconstruct with the ASTRA toolbox, how to change between different ASTRA reconstruction methods and between CPU and GPU implementations, and how to specify options for each method. Furthermore, an example was given where an ASTRA plugin was used to reconstruct the acquired data. For a specific dataset, we compared the computation time of various methods included in TomoPy and the ASTRA toolbox, which showed that the GPU-enabled iterative SIRT method of the ASTRA toolbox significantly reduced computation time compared with the CPU-based SIRT method of TomoPy. Finally, we computed reconstructions using different reconstruction methods for a single slice of experimental data, showing how ASTRA’s advanced reconstruction methods in combination with TomoPy’s advanced pre-processing methods can help reduce artifacts in reconstructions of tomographic synchrotron data, in particular in challenging scenarios where only a limited set of projections are available.

## Supplementary Material

Code for all scripts given in main paper. DOI: 10.1107/S1600577516005658/pp5084sup1.txt

## Figures and Tables

**Table 1:** List of tomographic reconstruction methods included in TomoPy and ASTRA for two-dimensional parallel-beam geometries For more information about the FP, BP, FBP, ART, SIRT and SART methods, we refer to Kak & Slaney (2001 ▸ ▸). For the Gridrec, MLEM, OSEM, PML and CGLS methods, we refer to Dowd *et al.* (1999 ▸), Dempster *et al.* (1977 ▸), Hudson & Larkin (1994 ▸), Chang *et al.* (2004 ▸) and Hansen (1998 ▸), respectively.

TomoPy				ASTRA		
Method	CPU	GPU		Method	CPU	GPU
ART	×			ART	×	
BART	×			BP	×	×
Gridrec	×			CGLS	×	×
MLEM	×			FP	×	×
OSEM	×			FBP	×	×
PML	×			MLEM		×
OSPML	×			SART	×	×
SIRT	×			SIRT	×	×

**Table 2:** List of common options that are used when reconstructing with the ASTRA toolbox through TomoPy

Option	Description	Example values
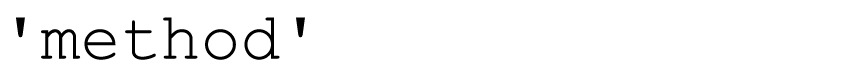	Which reconstruction method to use	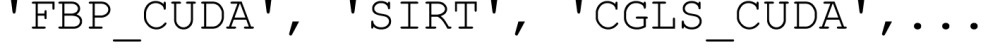
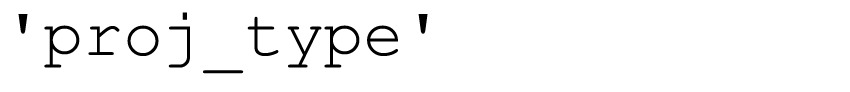	Which projection kernel to use	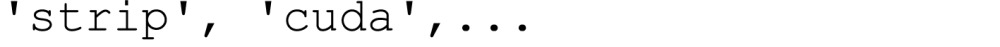
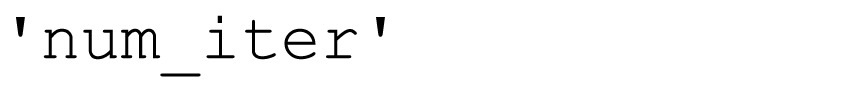	Number of iterations to use in iterative method	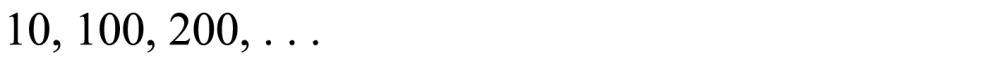
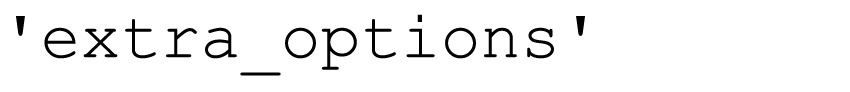	Python dictionary with extra method-specific options	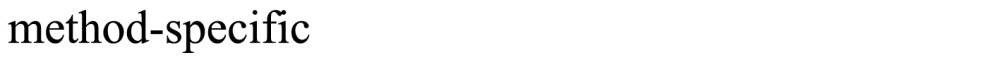
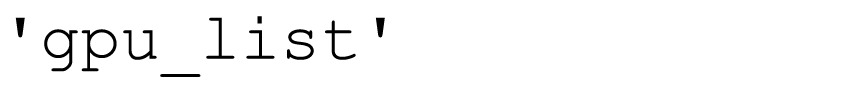	List of GPU indices to use for reconstruction